# Improving Accuracy for Image Fusion in Abdominal Ultrasonography 

**DOI:** 10.3390/diagnostics2030034

**Published:** 2012-08-27

**Authors:** Caroline Ewertsen, Kristoffer L. Hansen, Birthe M. Henriksen, Michael B. Nielsen

**Affiliations:** Department of Radiology, Rigshospitalet, Copenhagen Univsersity Hospital, Blegdamsvej 9, Copenhagen OE DK-2100, Denmark; E-Mails: lindskov@gmail.com (K.L.H.); birthehenriksen@gmail.com (B.M.H.); mbn@dadlnet.dk (M.B.N.)

**Keywords:** ultrasound, image-fusion, CT, accuracy

## Abstract

Image fusion involving real-time ultrasound (US) is a technique where previously recorded computed tomography (CT) or magnetic resonance images (MRI) are reformatted in a projection to fit the real-time US images after an initial co-registration. The co-registration aligns the images by means of common planes or points. We evaluated the accuracy of the alignment when varying parameters as patient position, respiratory phase and distance from the co-registration points/planes. We performed a total of 80 co-registrations and obtained the highest accuracy when the respiratory phase for the co-registration procedure was the same as when the CT or MRI was obtained. Furthermore, choosing co-registration points/planes close to the area of interest also improved the accuracy. With all settings optimized a mean error of 3.2 mm was obtained. We conclude that image fusion involving real-time US is an accurate method for abdominal examinations and that the accuracy is influenced by various adjustable factors that should be kept in mind.

## 1. Introduction

**Figure 1:**
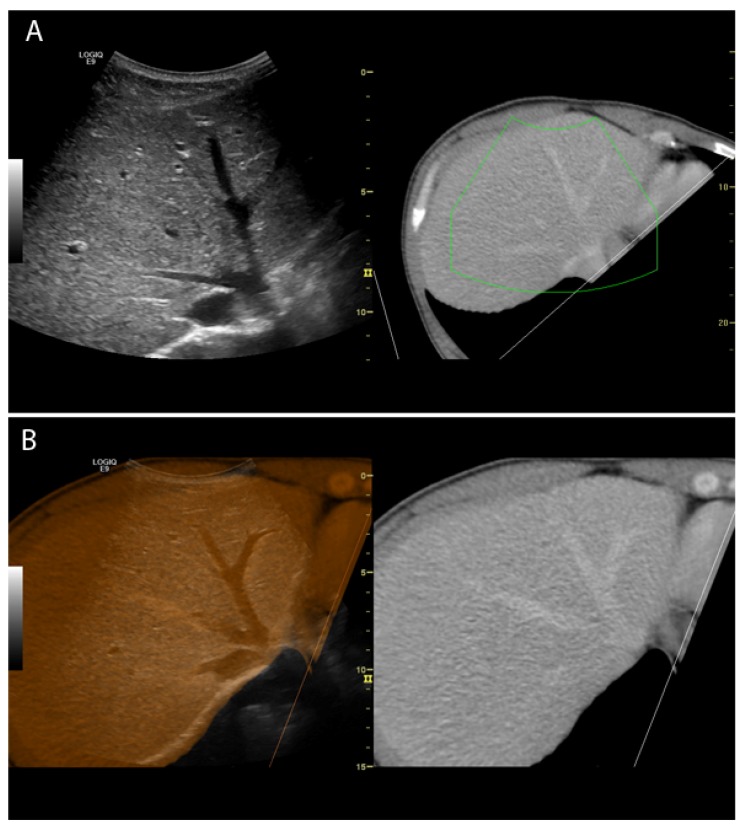
(**A**) Co-registered images of the liver shown side by side (ultrasound (US): **left**; computed tomography (CT): **right**); The green box indicates the scanning area (**B**) CT and US images of the liver overlaid (**left**) and corresponding CT-image (**right**).

Image fusion involving real-time ultrasonography (US) is a method, where previously recorded computed tomography (CT) or magnetic resonance images (MRI) are shown simultaneously with live US images, hereby enabling one to benefit from two imaging modalities in one examination. The images can be shown side by side or in one single image where the images are overlaid/fused (Figure 1). After an initial co-registration the CT or MRI images are reformatted in a projection to fit the live US images. Image fusion is based on software and a magnetic positioning system, and is implemented into several commercially available US systems (GE, Hitachi, Esaote, Philips). Obvious advantages in image fusion are the possibility of real-time guidance on lesions that are difficult to distinguish sonographically, and improving overview in areas with limited US visualization; this could be of abscesses containing air, lesions in the hepatic dome or lesions hidden behind ribs [1,2,3,4].

**Figure 2:**
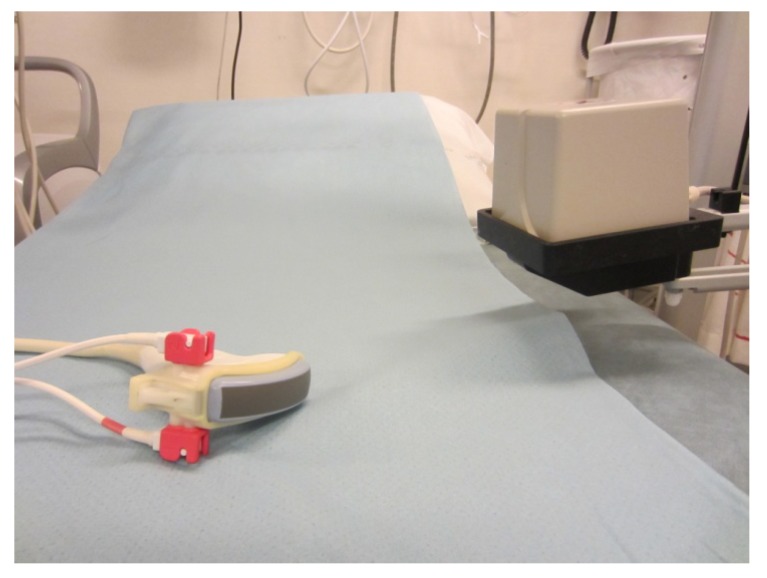
Magnetic positioning system. Magnetic sensors attached to the transducer (**left**) and magnetic transmitter (**right**).

When performing a fusion-guided US examination magnetic sensors are attached to the transducer and a magnet is placed beside the patient (Figure 2). The co-registration procedure is performed using common points or planes, which can be identified on both modalities. A minimum of three common points is necessary for the co-registration after an initial plane-lock where common planes are identified. Few studies have been performed regarding the accuracy of the co-registration procedure [5,6]. However, deformation of the liver during respiration has been described previously for MRI examinations [7].

The aims of our study were (1) to determine whether the accuracy depended on patient positioning and respiratory phase (2) to determine if the accuracy of the co-registration was dependent on the distance from the region of interest to the co-registration area using the optimal setting determined by the primary aim.

Our hypotheses were that accuracy depended on the co-registration being performed in the same respiratory phase as the CT examination more than on positioning of the arms. Furthermore, that the accuracy would decrease as the distance from the co-registration area increased.

## 2. Experimental Section

One normal-weight, healthy volunteer, who underwent CT examination during routine clinical practice, gave informed consent to participate in the study. One physician with more than 6 years experience of ultrasound performed, in a blinded trial, the US examinations.

### 2.1. Imaging

A LOGIQ E9 system (GE healthcare, Chalfont St. Giles) with incorporated software for fusion imaging and a convex-array C1-5 MHz transducer with abdominal setting was used. Two magnetic sensors were attached to the transducer and a magnetic transmitter was placed on the left side of the volunteer, in a distance of 5 cm from the skin. The previously recorded CT examination was loaded into the system from a compact disc.

An abdominal CT examination with and without contrast-enhancement was carried out on a Philips Brilliance 64 system (Philips Healthcare, Netherlands). The contrast agent was Omnipaque 350 mg/mL in a dose of 60 mL and water as oral contrast. The volunteer was scanned in the supine position with the arms stretched above the head with breath hold at deep inspiration. A contrast-enhanced series was chosen for the co-registration procedure in order to improve the delineation of the vessels.

### 2.2. Co-Registration

An initial plane lock was performed using the umbilicus as a common reference point, which will bring the images into a rough spatial alignment. Afterwards, 3 common points were used for the co-registration procedure. It was possible to add more points, which would theoretically improve the accuracy. However, in order to simplify the procedure only three points were chosen. The first point was the confluence of the hepatic veins into the inferior caval vein. The second point was the bifurcation of the left branch of the portal vein and the third point was a confluence of the middle hepatic vein in the right liver lobe. After each point was set, the images were aligned by the system. We repeated this co-registration procedure 20 times in four different settings: Arms above the head+deep inspiration, arms resting on the chest+deep inspiration, arms above the head+neutral respiration and arms resting on the chest+neutral respiration adding up to 80 co-registrations, in order to evaluate which parameters influenced the accuracy of the co-registration. The CT examination had been performed with the arms above the head and deep inspiration, thus the first setting mimicked this setting. After finishing each co-registration, the system was reset.

Afterwards, 20 co-registrations with the arms above the head and deep inspiration were performed to evaluate the misalignment in [mm] between corresponding points on CT and US images. These points were identified by the operator, and the misalignment was calculated with built-in software in the US system (GE Healthcare, Chalfont St Giles). The points were chosen in variable distances from the co-registration area to examine how the accuracy depended on this distance. The points were placed in four easy recognizable locations: the confluence of a hepatic vein in the left liver lobe (LL), the confluence of a hepatic vein in the right liver lobe (RL), the exit of the right renal artery (RA) from the aorta and the aortic bifurcation (AB). The distance between the co-registration area and the four locations was measured from the CT scan using the open-source OsiriX^®^ PAC software system.

### 2.3. Accuracy

Root Mean Square Deviation (RMSD) given in (mm) is a well-known method for measuring accuracy in image fusion, as it is the standard deviation of the mean distance between the corresponding co-registration points on CT and sonograms. After each co-registration the RMSD was automatically provided by the system. This was used to evaluate the accuracy of the co-registration in the four different settings, and thus determine if the accuracy depended on patient positioning and respiratory phase.

### 2.4. Statistics

Comparisons between the positions and respiratory phases in terms of measured RMSD were calculated using students’ t-test. To examine how the accuracy of corresponding points depended on distance to the co-registration area one-way analysis of variance (one-way ANOVA, df = 3) was performed. The distance to the co-registration area and the accuracy of the image fusion was investigated with simple linear regression. The statistical software SPSS 17.0 for windows (SPSS Inc.) and MATLAB 6.5 (Mathworks, Natick, MA, USA) were used for the calculations. The significance level was set at 0.05.

## 3. Results and Discussion

### 3.1. Results

**Figure 3:**
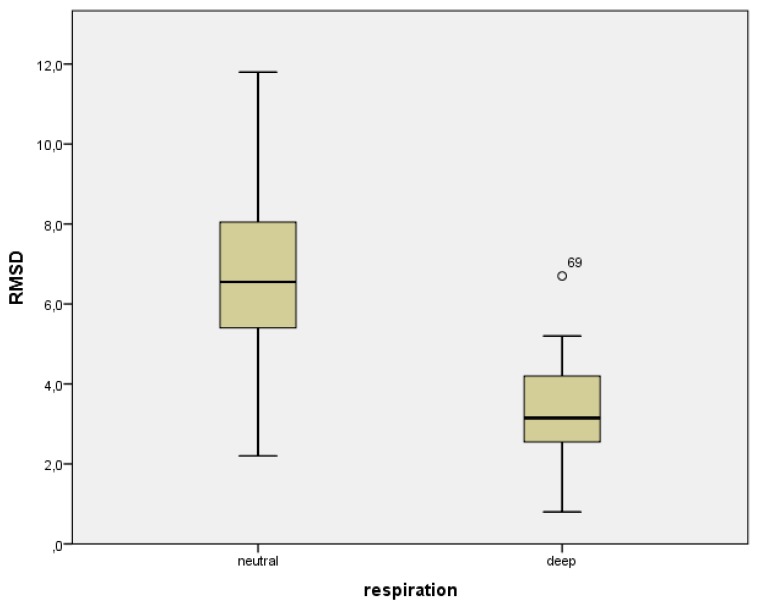
Box-whiskers plot showing distribution of accuracies for deep and neutral respiration, when arms were above the head.

A total of 20 co-registrations were made in four different settings, adding up to 80 co-registrations. A significant difference (p < 0.001) in RMSD was found between deep inspiration (3.2 mm (95%CI 2.6–3.8)) and neutral respiration (6.5 mm (95%CI 5.4–7.6)) when the arms were above the head (Figure 3). The difference was likewise significant (p < 0.001) between deep inspiration (3.5 mm (95%CI 2.9–4.1)) and neutral respiration (6.7 mm (95%CI 5.6–7.8)) when arms were on the chest. However, no significant difference was found in RMSD between the arm positions in deep inspiration (p = 0.43). 

**Figure 4:**
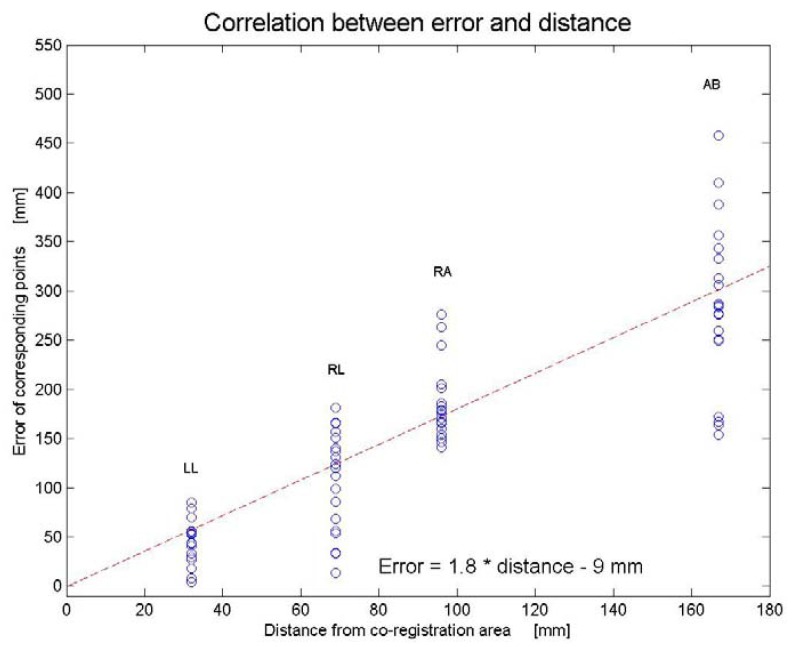
Correlation between error of corresponding points and distance to co-registration area. Line of best fit (dashed line) is drawn.

When evaluating the dependency of distance from the co-registration area, the volunteer was positioned with her arms above the head and breath-hold in deep inspiration. Using the same co-registration points as previously, 20 co-registrations were carried out. The distances between corresponding points, *i.e*., the error of alignment at the four locations as well as the distances from the co-registration area to the four locations (LL: 32 mm, RL: 69 mm, RA: 96 mm, AB: 167 mm) were registered. The distance between corresponding points increased with increasing distance to the co-registration area. The scatter plot showing the relation between alignment error and distance to the co-registration area is displayed in (Figure 4). 


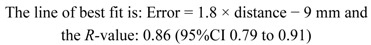
(1)

### 3.2. Discussion

We have successfully evaluated the accuracies in humans for fusion guided US in the liver, when varying breathing and patient positioning. By optimizing the co-registration procedure a mean accuracy of 3.2 mm for intraabdominal organs was obtained, which is confirmed by other groups. In patient studies on image fusion in the liver and on head and neck, the RMSDs have varied between 2.3 and 10.4 mm in previous studies [8,9,10,11]. Different transformation algorithms and varying methods of co-registration have been used in these studies.

In a more recent study different parameters as age of CT, general anesthesia or not, and landmarks for co-registration were varied and the accuracy of the co-registration was evaluated [6]. The best accuracy of 1.9 ± 1.4 mm was obtained when the examination was performed with the patient in general anesthesia and immediately after the CT procedure. However, this setting may be difficult to obtain.

We measured the accuracy as RMSD: a well-established method used when evaluating the accuracy of co-registered medical images [9,10,11,12,13].

Our results show a higher RMSD in humans than in phantom studies, because it is more difficult to precisely identify and mark the same points or planes [9]. As our results show, this is due to patient positioning and respiratory movements. Body Mass Index (BMI), insonating angle, operator experience, the distance between magnet and sensor for the magnetic positioning system and metal in the bed may also influence the accuracies [14].

In PET/CT where the images are recorded almost simultaneously and fusion carried out automatically, the reported accuracy is less than 2 mm in a phantom and less than 4 mm in patients [8]. Thus, real-time fusion involving US appears equally accurate to static fusion systems.

Furthermore, we showed that the accuracy of the co-registration is dependent on the distance between the region of interest and the co-registration area (*R* = 0.86), measured as misalignment between corresponding points. According to the equation for the line of best fit (Figure 3), the error is related to the distance from the co-registration area with a factor 1.8. Therefore, aligning corresponding points 10 mm from the co-registration area results, on average, in a misalignment of 18 mm. Thus, we were able to confirm both our hypotheses.

The strength of our study is that it focuses only on the accuracy of the co-registration procedure of fusion-guided US in a human model. All co-registrations were performed in the same healthy volunteer in order to minimize confounding from BMI, patient co-operation and quality of CT-data. The number of co-registrations was large. We measured the distance from the co-registration area in the liver to the measuring points in the second part of the study in order to calculate the correlation between accuracy and distance from co-registration area. This showed a large decrease in accuracy when the distance from the co-registration area increased.

A weakness of the study is that only one normal-weight volunteer, who could easily cooperate was examined, and thus, the setting was optimal. We would expect less accurate co-registrations in more obese patients because of the increased pressure of the transducer in order to obtain acceptable images. In such patients the accuracy could possibly be improved by adding more co-registration points and by checking the alignment continuously by using electronic markers on both images.

Another weakness is the inevitable inaccuracy when marking points in neutral respiratory phase, thus, the accuracy of this series could possibly be improved if the volunteer had held her breath when marking the points. Furthermore, only one operator performed the image fusion, thus, inter-observer differences have not been assessed in this study.

## 4. Conclusions

In conclusion, we believe fusion guided ultrasound is a feasible and accurate examination. However, the alignment of the images may be improved by using the same respiratory phase as in the previous examination (CT or MRI), positioning the patient as in the CT or MRI setting and performing the co-registration in proximity to the area of interest. 
